# HIF Oxygen Sensing Pathways in Lung Biology

**DOI:** 10.3390/biomedicines6020068

**Published:** 2018-06-06

**Authors:** Andrés A. Urrutia, Julián Aragonés

**Affiliations:** 1 Research Unit, Hospital of Santa Cristina, Research Institute Princesa (IP), Autonomous University of Madrid, 28009 Madrid, Spain; 2 CIBER de Enfermedades Cardiovasculares, Carlos III Health Institute, 28029 Madrid, Spain

**Keywords:** lungs, HIF, oxygen, pulmonary hypertension, hypoxia, airway, endothelium, pulmonary smooth muscle cells

## Abstract

Cellular responses to oxygen fluctuations are largely mediated by hypoxia-inducible factors (HIFs). Upon inhalation, the first organ inspired oxygen comes into contact with is the lungs, but the understanding of the pulmonary HIF oxygen-sensing pathway is still limited. In this review we will focus on the role of HIF1α and HIF2α isoforms in lung responses to oxygen insufficiency. In particular, we will discuss novel findings regarding their role in the biology of smooth muscle cells and endothelial cells in the context of hypoxia-induced pulmonary vasoconstriction. Moreover, we will also discuss recent studies into HIF-dependent responses in the airway epithelium, which have been even less studied than the HIF-dependent vascular responses in the lungs. In summary, we will review the biological functions executed by HIF1 or HIF2 in the pulmonary vessels and epithelium to control lung responses to oxygen fluctuations as well as their pathological consequences in the hypoxic lung.

## 1. Introduction

The lungs are responsible for oxygen uptake and the pulmonary vascular responses that ensure adequate blood oxygenation in response to low oxygen tensions. These responses can be initiated in acute conditions of hypoxia (seconds to minutes), involving mechanisms such as membrane depolarization and the influx of calcium into pulmonary artery smooth muscle cells (PASMCs), which leads to elevated pulmonary vascular tone in poorly oxygenated alveoli [1,2,3]. In more prolonged hypoxic conditions (hours to days), the lungs also initiate a response through the hypoxia-inducible factors (HIFs) [4,5].

HIFs are heterodimeric transcription factors and comprised of one HIFα subunit (HIF1α, HIF2α or HIF3α) and a member of the HIFβ family, also known as the aryl hydrocarbon receptor nuclear translocator (ARNT) [6]. While the HIFβ subunit is stable, the stability of the HIFα subunits fluctuates in response to changes in oxygen tension through the prolyl 2-oxoglutarate-dependent Fe^2+^-dioxygenases PHD1, PHD2, and PHD3 [7,8,9]. In normoxic conditions, PHDs use oxygen to hydroxylate two conserved proline residues in the HIFα subunits and these hydroxylated prolyl residues can be recognized by the VHL (von Hippel-Lindau)/E3 ubiquitin ligase complex, which leads HIFα subunits to proteasomal degradation [9,10]. However, in hypoxic conditions, there is insufficient oxygen for PHDs to hydroxylate the HIFα subunits, which preclude their recognition by VHL leading to HIFα subunits stabilization. Consequently, HIFα subunits can enter the nucleus where they heterodimerize with HIFβ subunits and bind to DNA at hypoxia response elements (HREs), thereby driving a HIF-dependent transcriptional program [11,12].

While this molecular machinery is certainly operative in lung tissue [13,14,15], it has been less explored here than in other biological scenarios, which is surprising considering the fundamental role of the lungs in oxygen homeostasis. In conditions of hypoxia, both HIF1α and HIF2α are stabilized in the lungs [13,14,15]. In particular, Wiesener et al. showed that—in contrast to other tissues such as liver or kidney—pulmonary HIF2α expression is induced in hypoxia in a more larger extent than upon exposure to CO [14], which suggests that HIF activation in the lungs (possibly mainly in the airway epithelium) largely relies directly on the oxygen supply in the airway more than on red blood cell oxygen delivery.

In this review we focus on recent in vivo studies that have shed new light on the role of HIF1α and HIF2α isoforms in the response of the pulmonary vascular and airway epithelium to oxygen fluctuations (hypoxia).

## 2. The HIF Oxygen Sensing Pathways in the Pulmonary Vasculature

A well-recognized physiological response to acute or sustained hypoxia is the constriction of small pulmonary arteries, which is driven by hypoxia-dependent pathways in pulmonary artery smooth muscle cells (PASMCs) and endothelial cells [1,2,16,17,18]. In the lung, this physiological mechanism (termed hypoxic pulmonary vasoconstriction) has evolved to restrict blood flow to the alveoli that are less oxygenated, redirecting the blood towards the better oxygenated ones. However, when these hypoxic conditions become chronic and systemic, and when the oxygenation of virtually every alveolus is compromised, this physiological response becomes pathological and results in sustained pulmonary hypertension (PH). This pathological scenario is defined as an increase in the mean pulmonary arterial pressure above 25 mmHg at rest [19]. PH is characterized by increased smooth muscle coverage (muscularization) of the pulmonary arteries, with a subsequent increase in pulmonary vascular resistance that ultimately leads to right ventricular (RV) hypertrophy and heart failure [1]. PH can be initiated in different pathological settings and hypoxia-dependent PH has been specifically categorized as “pulmonary hypertension due to lung diseases and/or hypoxia” [20,21].

### 2.1. Chronic Activation of the HIF Pathways Leading to Pulmonary Hypertension

A large body of evidence has shown that sustained environmental hypoxia can induce PH through the HIF pathway. Indeed, the first evidence of the role of HIF in PH in vivo came from HIF-deficient animals. Mice carrying a heterozygous germline deletion of *Hif1a* or *Hif2a* exposed to chronic hypoxia showed impaired PH development, in part due to the more limited pulmonary vascular remodeling [16,17]. Further evidence in humans and mice about the role of HIF signaling in PH come from studies about the pathological inherited mutations resulting in HIF overactivation, which leads to PH. Indeed, homozygosity for the VHL allele harboring the mutation R200W, which degrades HIFα subunits less efficiently than the WT VHL, is associated with HIF2α-dependent polycythemia and PH [22]. Moreover, patients and mice carrying a gain of function HIF2α mutation (G537W in human or G536W in mice) develop severe PH, as well as exacerbated erythrocytosis [23,24].

These initial studies highlighted the contribution of the HIF pathway to hypoxia-induced hypertension. As discussed below, more recent in vivo studies have provided new insights into the relative contribution of HIF1α and HIF2α isoforms in different pulmonary cell types and their specific contribution to PH, as well as identifying the downstream HIF-dependent events relevant to this pathological scenario.

### 2.2. The HIF1α Activity in Pulmonary Smooth Muscle Cells Associated with Hypoxia-Induced Pulmonary Hypertension

Although the seminal studies indicated above provided evidence of the role HIF in PH, until recently the contribution of specific lung cell types to these responses remained unclear. In the last few years, transgenic animals targeting specific cell types has unveiled the role of HIF isoforms in PH. Pulmonary vascular remodeling is considered necessary for the development of hypoxia-induced PH and it involves the appearance of smooth muscle-like cells in vessels of the alveolar wall that is not normally muscularized, as well as the medial and adventitial thickening of the muscle and elastic vessels [25]. The main cell types involved in hypoxia driven vascular remodeling under are thought to be PASMCs and endothelial cells, although the role of other cells like pericytes, fibroblasts, macrophages or airway epithelial cells cannot be ruled out.

**Figure 1:**
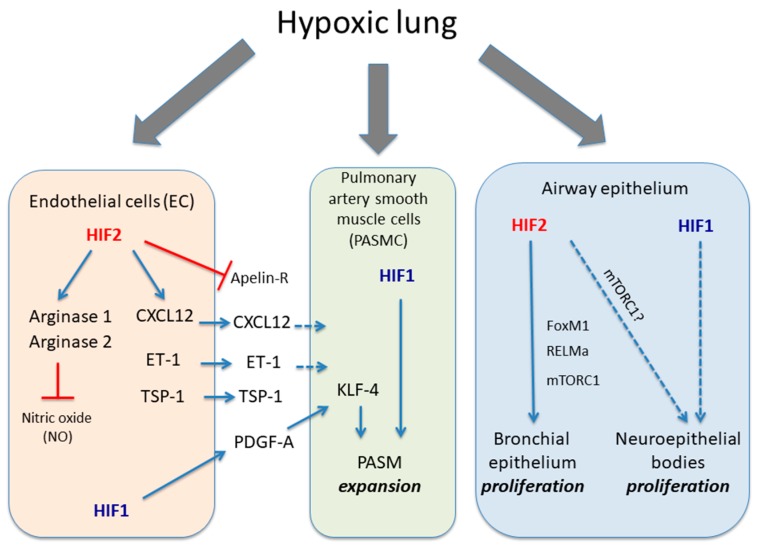
Role of HIF1α and HIF2α isoforms in different lung cell types in response to hypoxia. The figure shows the biological actions executed of HIF1α and HIF2α isoforms in endothelial cells, PASMC and airway epithelium in response to hypoxia. Endothelial HIF2 induces a series of responses leading to vasoconstriction such as induction of arginase activity, which limit endothelial NO availability as well as release CXCL12, ET-1 and TSP1 in parallel with an inhibition of apelin receptor. HIF1 activity in endothelial cells can also release PDGF-B, which cooperates through KLF4 with PASMC HIF1 activity to promote their expansion. The figure also shows HIF2 activity in bronchial epithelial proliferation through potential mediators such as mTORC1, RELMα or FOXM1 as well as the potential role of HIF1α in neuroepithelial bodies proliferation located in pulmonary epithelium. Solid dark blue lines indicate activatory pathways; dashed dark blue lines indicate potential activatory actions and red lines indicate inhibitory actions.

Although the role of HIF in PASMCs and endothelial cells has not been thoroughly explored in vivo, the current experimental data seems robust enough to conclude that HIF activity in endothelial and PASMC are key to understand hypoxia-induced vascular remodeling and PH (Figure 1). Interestingly, Yu et al. showed that cultured PASMCs can express some detectable HIF1α signal under normoxia, which can be further enhanced in hypoxia [13]. The role for HIF1 in PASMCs in the vascular remodeling, muscularization and PH has been found upon *Hif1a* gene inactivation in SMCs using *Mhy11-CreERT2* mouse line [26]. Surprisingly, the attenuation of vascular remodeling and PH was not reflected by an attenuation of right ventricle cardiac hypertrophy, which is in line with previous data showing that increased PH might not fully explain right heart failure [27,28]. An additional study has used *Pdgfrb-CreERT2* mice in order to inactivate *Hif1a* in previously identified pulmonary PDGFRβ^+^/αSMC^+^ progenitors that are primed in hypoxia-induced PH [29,30]. These mice show that distal arterioles are less covered by α-smooth muscle positive cells and a profound attenuation of PH and right ventricular hypertrophy [29]. The differences between these two studies can arise by the different Cre lines employed. In this line, *Pdgfrb-CreERT2* can inactivate *Hif1a* not only in those pulmonary PDGFRβ^+^/αSMC^+^ progenitors but also in other mural populations such as heart PDGFR-β^+^ pericytes that could also contribute to HIF1α-dependent RV hypertrophy [29]. Finally, another study have revealed that *Hif1a* inactivation in PASMCs using *SM22-Cre* mice also shows a trend towards reduced artery muscularization when these mice are exposed to hypoxia [31]. Yet surprisingly, a parallel role of HIF1α in reducing vascular tone by repressing myosin light chain phosphorylation was also shown [31]. In summary, HIF1 is clearly a driver of hypoxia-induced PH and the size and features of this effect may vary depending on the Cre line use, and which other cells different than PASMC can be targeted. Finally, it will also be crucial to study whether HIF2 activity in PASMCs also contributes to hypoxia-induced PH, which as yet remains unexplored.

### 2.3. The Endothelial HIF2 Pathway in Hypoxia-Induced Pulmonary Hypertension

As indicated previously, global *Hif2a* inactivation supports its role in hypoxia-induced PH [17]. More recent studies revealed that the relevance of HIF2α in pulmonary vascular remodeling depends on its expression in endothelial cells [32,33,34,35] (Figure 1). Specific inactivation of *Hif2a* in endothelial cells using the *Cdh5* (VE-Cadherin)-*Cre* mouse line was sufficient to block the development of hypoxia-induced PH [32]. Furthermore, inactivation of the *Phd2* gene in endothelial cells using the same mouse Cre line (mimicking PHD2 inactivation in hypoxia) promoted HIF1α and HIF2α stabilization under normoxic conditions. More importantly, the loss of *Phd2* was sufficient to enhance pulmonary muscularization and right ventricular systolic pressure (RVSP), leading to right ventricular hypertrophy and premature death [32]. While *Hif1a* inactivation had no effect over the aforementioned phenotype, simultaneous *Hif2a* inactivation rescued PH and right ventricular hypertrophy in endothelial *Phd2* knockout mice. Similar results were obtained using a different mouse line expressing Cre recombinase under the control of the *Tie2* promoter [33]. Furthermore, when *Hif2a* but not *Hif1a* is specifically deleted in pulmonary endothelial cells using the L1-*Cre* mouse line, the development of hypoxia-induced PH and RV hypertrophy was abrogated [34]. In summary all these studies point out an essential role of endothelial cell HIF2 activity in hypoxia-induced PH. However, a recent study have shown that *Hif1a* gene inactivation in endothelial cells using Cdh5 (VE-Cadherin)-*Cre* mouse line can compromise hypoxia-induced PH [29]. This study proposes that HIF1 activity induces PDGF-B in endothelial cells that ultimately can impact on PASMC cells to promote KLF14-dependent PASMC expansion (Figure 1). The reasons of this discrepancy of this study is unclear and possibly could be related differences between hypoxia exposure and PHD2 inactivation but this certainly warrants further investigation.

These studies also explored possible HIF2-target genes in endothelial cells that might be responsible for the role of endothelial HIF2 in hypoxia-induced PH. Several mechanisms have been proposed to explain the role of HIF2 in pulmonary vasculature remodeling and hypertension. A well-recognized HIF2-dependent gene in lung tissue is endothelin-1 (*ET-1*) [17,22,23,24], which is a potent vasoconstrictor [36]. Indeed, ET1 expression is markedly elevated in response to hypoxia in a HIF2-dependent manner, as well as in mice harboring a human HIF2α gain-of-function mutation and in a model of Chuvash disease [17,24,37]. More recently, endothelial HIF2 was shown to be responsible not only for an elevation in ET-1 but also for the concomitant downregulation of the apelin receptor that mediates apelin-dependent vasodilatation [32,38]. ET-1 and apelin receptors have been identified in PASMC [39,40], and therefore these data suggest that endothelial HIF2 triggers different mechanisms that—in a paracrine manner—potentially favor PASMC-mediated vasoconstriction. An additional proposed mechanism based on the crosstalk between endothelial cells and PASMCs is the secretion of endothelial CXCL12, which is released from endothelial cells upon HIF2-activation and promotes the expansion of PASMCs [33]. Importantly, increased RVSP and RV hypertrophy in endothelial *Phd2*-deficient mice (see above) is abrogated when *Phd2* and *Cxcl12* are simultaneously inactivated in endothelial cells. On the other hand, Cowburn et al. observed that mutant mice with a conditional deletion of *Hif2a* in pulmonary endothelial cells showed a decreased expression of the enzymes Arginase 1 and 2 [34]. As these enzymes have been implicated in promoting vascular remodeling by reducing airway nitric oxide availability [41,42], it was hypothesized that HIF2-dependent arginase activity was involved in hypoxia-induced PH. Indeed, Arginase 1 gene inactivation in the pulmonary endothelium of animals exposed to chronic hypoxia reduces RVSP and RV hypertrophy development [34]. Interestingly, Cowburn also analyzed endothelial progenitor cells from PAH patients, which show increased arginase expression levels that can be further enhanced by hypoxia [34,42,43], suggesting that PH patients can develop a more robust vascular response to hypoxia. Finally, a recent study also showed that HIF2 induces the thromboposdin-1 (TSP-1) expression in hypoxic lung tissue [44], which can also contribute to PH given that TSP1 deficient mice are partially protected from hypoxia-induced pulmonary artery muscularization [45,46]. As mentioned previously, these mechanisms are not necessarily mutually exclusive. Indeed, it is tempting to think that the most likely scenario is one where all of the aforementioned mechanisms are implicated together, and that they synergize and cooperate to promote HIF2-dependent PH.

In summary, preclinical data suggest that HIF1 is the main player in PASMCs, favoring hypoxia-induced PH, while the relevance of HIF2 in this pathological lung scenario is mediated by its effects on endothelial cells-induced vascular constriction.

## 3. Role of HIF Pathway in the Airway Epithelium

As can be seen above, the relevance of the HIF pathway in lung biology has largely been studied in the pulmonary vascular bed (endothelial cells and pulmonary vascular SMCs), although the relevance of this pathway in airway biology has also been shown. Seminal studies initially show that HIF2α is essential for lung development and for airway biology in particular. Indeed, *Hif2a* deficient mice succumb to a respiratory distress syndrome in which the mice become cyanotic as a consequence of failed lung expansion. At the molecular level, HIF2α governs airway surfactant expression by regulating the vascular endothelial growth factor (VEGF-A), which ultimately controls alveolar epithelium maturation [47]. However, the responses to HIF signaling in the hypoxic airway epithelium in adulthood remain largely unknown, although there is some evidence that HIF influences proliferation in the airway and provides protection from hypoxia.

### The HIF Pathway in Airway Epithelium Proliferation

Although the role of HIF signaling has been mainly been explored in pulmonary vascular function, several studies have also highlighted the importance of HIF1α and HIF2α isoforms in the pulmonary epithelium. Immunohistological analysis showed nuclear HIF2α expression in the bronchial epithelial cells of hypoxic mice (10% O_2_) where induces the expression of well-recognized HIF2α-dependent genes in lung tissue, such as resistin-like α (RELMα, also named HIMF or FIZZ1) [48,49]. Human RELMβ (RELMα in mice) is a soluble factor that has been identified as a mitogenic factor for bronchial epithelial cells in vitro [50]. Indeed, HIF2 induces marked proliferation of bronchial epithelial cells after exposure to hypoxia (3–4 days), mainly in club cells [49] and possibly via RELMα. However, HIF2-dependent club cell proliferation may also be governed by cell autonomous mechanisms. Indeed, our own studies have shown that HIF2 acts as an activator of the mammalian target of rapamycin complex 1 (mTORC1) in clear cell renal cell carcinoma (ccRCC). The mTORC1 functions as a serine/threonine kinase that responds to amino acid availability and the energy status of the cell, playing a central role in cell growth and proliferation [51,52,53]. In fact, HIF2α-dependent mTORC1 activation is driven by the amino acid carrier SLC7A5 (also named LAT-1), provoking ccRCC proliferation [51]. We have also shown that this HIF2-Slc7a5-mTORC1 axis is operative in bronchial epithelial cells, suggesting its role in hypoxia-induced club cell proliferation [49,51]. Furthermore, HIF2 also induces the expression of FOXM1, a transcription factor that is critical for club cell proliferation and differentiation [54,55]. Overall, these data suggest that HIF2 fosters bronchial epithelial cell proliferation in hypoxia through cell autonomous mechanisms like mTORC1 and FOXM1 activation, as well as non-cell autonomous mechanisms such as those driven possibly by RELMα.

Cell proliferative responses to hypoxia are not restricted to club cells in the bronchial epithelium; they have also been found in the pulmonary neuroepithelial bodies (NEBs). These structures are presumed airway sensors, made-up of innervated clusters of amine (serotonin) and peptide-producing cells that reside in the bronchial epithelium [56,57]. In humans and in animal models, hypoxia leads to hyperplasia of NEBs, and PHD1 and PHD3 have been implicated in this response [56,58,59]. Indeed, *Phd1*- and *Phd3*-deficient mice show marked hypertrophia and NEB proliferation, in parallel to the induction of HIF1α in some of synaptophysin (SYP) positive cells typically found in NEBs [56,59]. However, it is unclear whether or not HIF1 contributes to NEB proliferation in response to hypoxia and whether NEB hyperproliferation is driven by HIF2, as in club cells. Nevertheless, NEBs are presumed to be polymodal airway sensors analogous to the carotid body (CB) glomus cells [3,60] and recently, the HIF2-mTORC1 proliferative pathway was shown to be involved in chronic hypoxia-induced enlargement of the carotid body (CB) [61,62]. Hence, the HIF2-mTORC1 pathway might not only drive hypoxia-dependent CB and club cell proliferation but also, hypoxia-dependent NEB hyperplasia. Finally, it should also be considered that CB enlargement and NEB hyperplasia in response to hypoxia are currently thought to be adaptive responses to hypoxia, potentiating ventilatory responses. Therefore, it will be relevant to understand the physiological meaning of this additional response involving HIF2α-dependent bronchial epithelium proliferation and how this might be integrated in the context of the adaptive response to systemic hypoxia. Moreover, further experimentation in human pathophysiological scenarios will be necessary to unveil clinically relevant aspects of the HIF oxygen-sensing pathway in human airway biology.

Finally, it might be considered that HIF2-dependent actions in bronchial epithelium could contribute not only for local airway adaptation to hypoxia, but also to more distant pulmonary vessel remodeling described above. In this line, hypoxia-induced RELM-α (largely produced in bronchial epithelium) causes PH characterized by an increase in mean pulmonary artery pressure, pulmonary vascular resistance, right heart hypertrophy, and vascular remodeling caused by chronic hypoxia as well as lung vascular inflammation [63,64]. At molecular level, RELMα also recruits bone marrow-derived macrophages, promotes IL-6 expression in PASMC and macrophages through HIF1α and favors vascular remodeling associated to PH [63,64]. These data suggest a possible HIF2α-dependent communication between airway epithelium and vascular bed, which can also contribute to HIF-dependent vascular remodeling and PH in the hypoxic lung.

## 4. Concluding Remarks and Perspectives

In this review we have focused on the HIF1 and HIF2-dependent responses that are confined to the pulmonary vascular bed and airway epithelium. However, it should be noted that some of the genes induced by the HIF pathway are soluble factors that may be implicated in vascular and epithelium communication. As such, HIF2-dependent soluble factors released by the airway epithelium (i.e., RELMα) may act in synergy with other effectors of HIF2 in endothelial cells to drive pathological vascular remodeling in PH. Inactivation of the *Hif2a* gene in different cell types in the airway epithelium will help to understand the contribution of local airway responses driven by HIF2 to distant vascular remodeling in PH. Moreover, many of the studies into the role of HIFs in lung biology have focused on pathological PH in chronic hypoxia (weeks and months). Thus, a greater effort to explore the short-term HIF-dependent responses to hypoxia (possibly non-pathological) will be necessary to define the novel molecular and cellular adaptive responses to pulmonary oxygen fluctuations.
